# Changes in transcriptome of macrophages in atherosclerosis

**DOI:** 10.1111/jcmm.12591

**Published:** 2015-05-13

**Authors:** Dimitry A Chistiakov, Yuri V Bobryshev, Alexander N Orekhov

**Affiliations:** a Department of Medical Nanobiotechnology, Pirogov Russian State Medical UniversityMoscow, Russia; b Faculty of Medicine and St Vincent's Centre for Applied Medical Research, University of New South WalesSydney, NSW, Australia; c School of Medicine, University of Western SydneyCampbelltown, NSW, Australia; d Institute for Atherosclerosis Research, Skolkovo Innovative CenterMoscow, Russia; e Laboratory of Angiopathology, Institute of General Pathology and Pathophysiology, Russian Academy of SciencesMoscow, Russia; f Department of Biophysics, Biological Faculty, Moscow State UniversityMoscow, Russia

**Keywords:** macrophages, phenotypic heterogeneity, transcriptome, atherosclerosis, atherogenesis

## Abstract

Macrophages display significant phenotypic heterogeneity. Two growth factors, macrophage colony-stimulating factor and chemokine (C-X-C motif) ligand 4, drive terminal differentiation of monocytes to M0 and M4 macrophages respectively. Compared to M0 macrophages, M4 cells have a unique transcriptome, with expression of surface markers such as S100A8, mannose receptor CD206 and matrix metalloproteinase 7. M4 macrophages did not express CD163, a scavenger receptor for haemoglobin/haptoglobin complex. Depending on the stimuli, M0 macrophages could polarize towards the proinflammatory M1 subset by treatment with lipopolysaccharide or interferon-γ. These macrophages produce a range of proinflammatory cytokines, nitric oxide, reactive oxygen species and exhibit high chemotactic and phagocytic activity. The alternative M2 type could be induced from M0 macrophage by stimulation with interleukin (IL)-4. M2 macrophages express high levels of CD206 and produce anti-inflammatory cytokines IL-10 and transforming growth factor-β. M1, M2 and M4 macrophages could be found in atherosclerotic plaques. In the plaque, macrophages are subjected to the intensive influence not only by cytokines and chemokines but also with bioactive lipids such as cholesterol and oxidized phospholipids. Oxidized phospholipids induce a distinct Mox phenotype in murine macrophages that express a unique panel of antioxidant enzymes under control of the redox-regulated transcription factor Klf2, resistant to lipid accumulation. In unstable human lesions, atheroprotective M(Hb) and HA-mac macrophage subsets could be found. These two subsets are induced by the haemoglobin/haptoglobin complex, highly express haeme oxygenase 1 and CD163, and are implicated in clearance of haemoglobin and erythrocyte remnants. In atherogenesis, the macrophage phenotype is plastic and could therefore be switched to proinflammatory (*i.e*. proatherogenic) and anti-inflammatory (*i.e*. atheroprotective). The aim of this review was to characterize changes in macrophage transcriptome in atherosclerosis and discuss key markers that characterize different phenotypes of macrophages present in atherosclerotic lesions.

- Introduction
- Monocyte–macrophage differentiation
- Macrophage polarization
- Macrophage phenotypes in atherosclerosis
- Changes in macrophage transcriptome during differentiation and polarization
  Monocyte–macrophage differentiation
  ‘Classical’ polarization of M0 macrophages to M1
  ‘Alternative’ polarization of M0 macrophages to M2
  CXCL4-induced M4 macrophages
  Cholesterol load
  Mox macrophages
- Conclusion

## Introduction

**Figure 1:**
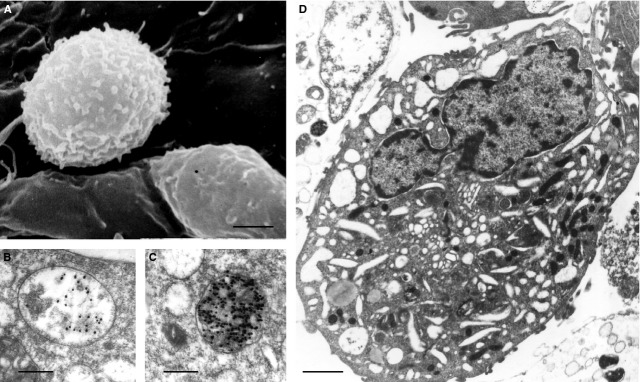
Adhesion of monocytes to the luminal endothelium that covers a fatty streak (A) and the accumulation of oxidized lipoproteins of low density (oxLDL) in macrophages in an *in vitro* experiment (B and C); incubation of macrophages with oxLDL is accompanied by the formation of foam cells (D) 5. (A) Scanning electron microscopy. (B and C) Oxidized LDL were labelled with gold particles. (D) Transmission electron microscopy. Scale bars = 3 μm (A), 250 nm (B and C), 1 μm (D).

Atherosclerosis is characterized by accumulation of modified serum lipids and lipoproteins in the arterial intima 1. Blood monocytes respond to the subendothelial lipid deposits by increased adhesion to the luminal endothelium (Fig.1A), followed by the penetration through the endothelium into the tunica intima where monocytes differentiate to macrophages and try to clear modified lipoproteins 1–7. As shown in *in situ* and *in vitro* studies, macrophages that intensely engulf modified lipoproteins become uploaded with lipids, and this leads to the formation of foam cells (Fig.1B–D) 1–7. These macrophages terminally lack the ability to emigrate from the initial lesion 8. Indeed, they contribute to aggravating atherosclerotic inflammation and formation and progression of the atherosclerotic plaque 9. The macrophages could be found in various lesion regions and in the vicinity to the lesion including plaque shoulder, the next zone to the necrotic core of the plaque and/or the next zone to the calcified or rigid arterial regions 10. In atherosclerosis, macrophages become exposed to various microenvironmental stimuli, mostly to proinflammatory signals, which drive their activation and polarization. The differentiation of monocytes to macrophages is likely to be terminal, but the macrophages have a plasticity to change a phenotype in response to incoming stimuli.

## Monocyte–macrophage differentiation

Macrophage differentiation means the differentiation of monocytes to macrophages when monocytes infiltrate into the arterial wall and transform from round-shaped cells to irregularly shaped cells capable to intake antigens and migrate within the wall. *In vitro*, monocyte–macrophage differentiation induced by macrophage colony-stimulating factor (M-CSF) which mostly completes within the first 3 days 11. M-CSF is the best studied factor that induces differentiation of monocytes to macrophages. The treatment of cultured monocytes with M-CSF results in reduction in expression of the surface marker CD14 and the induction of macrophage-specific markers such as CD68 11. The proatherogenic role of M-CSF and its receptor was shown in apolipoprotein E (ApoE)-deficient mice, a murine strain susceptible to atherosclerosis. The deletion of genes encoding M-CSF and its receptor led to significantly diminished atherosclerosis in ApoE-deficient mice 12,13. M-CSF drives differentiation of monocytes to naïve M0 macrophages and anti-inflammatory M2a subset of macrophages 14.

Monocyte–macrophage differentiation could be also induced by granulocyte–macrophage colony-stimulating factor (GM-CSF) and chemokine (C-X-C motif) ligand 4 (CXCL4, or platelet factor 4). However, these growth factors are inducible, whereas M-CSF is constantly circulates in the blood 14. GM-CSF induces differentiation of monocytes to proinflammatory M1 macrophages 15, whereas CXCL4 primes monocyte differentiation to M4 cells, a specific subset of macrophages 16.

## Macrophage polarization

**Figure 2:**
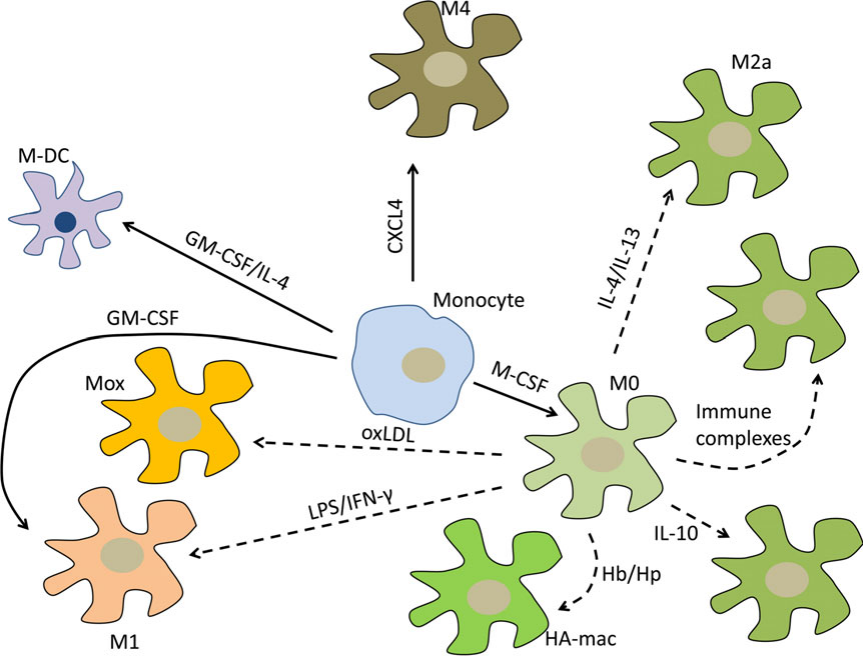
Phenotypes of macrophages in atherosclerotic lesions. In the subendothelial layer, monocytes could differentiate to various macrophage subtypes depending on the microenvironmental stimuli. Solid arrows indicate monocyte–macrophage differentiation, whereas dotted arrows indicate macrophage polarization. M-DC: dendritic cells; Mox: foam cells induced by oxidized low-density lipoproteins (oxLDL); HA-Mac: haemorrhage-associated macrophages; CXCL4: chemokine (C-X-C motif) ligand 4; IL: interleukin; IFN-γ: interferon-γ; LPS: lipopolysaccharide; M-CSF: macrophage colony-stimulating factor; GM-CSF: granulocyte–macrophage colony-stimulating factor.

Polarization of macrophages designates their plasticity, *e.g*. ability to switch phenotype and functional characteristics in response to external signals. The ‘classically polarized’ M1 macrophages could be induced from M0 macrophages by lipopolysaccharide (LPS) or interferon-γ (IFN-γ) 17 (Fig.2; Table1). The ‘alternatively polarized’ M2 macrophages could be induced by interleukin-4 (IL-4) 18. Within the anti-inflammatory M2 set, several subsets are distinguish depending on the polarizing stimuli such as M2a, M2b and M2c that could be induced from M0 by IL-4/IL-13, immune complexes + IL-1β or LPS, and IL-10/transforming growth factor (TGF)-β/glucocorticoids respectively 19. Briefly, M1 macrophages show proinflammatory properties because they produce a range of inflammatory cytokines such as IL-1, IL-6, IL-8, IL-12, IL-23, and tumour necrosis factor (TNF)-α, reactive oxygen species and nitric oxide, whereas M2 macrophages are rather anti-inflammatory as they could be induced by Th2 cytokines and produce IL-10 and various scavenger receptors such as CD36, macrophage scavenger receptor 1, macrophage receptor with collagenous structure and mannose receptor (MRC1 or CD206) 20. M1 macrophages are involved in management of Th1-dependent proinflammatory immune responses.

**Table 1:** Macrophage phenotypes observed in humans and mice*

Phenotype	Induction	Cell markers	Cytokines, chemokines and other secreted factors	Functions and properties	Presence in the plaque
M1	IFN-γ, TNF-α, LPS	IL-1β, IL-6, IL-12, IL-23, TNF-α, CXCL9, CXCL10, CXCL11, Arg-2 (mouse)	IL-6, IL-10^low^, IL-12^high^, IL-23, TNF-α, iNOS, ROS	Inflammatory response	Human, mouse
M2a	IL-4, IL-13	Human: CD206, IL1RN; Mouse: Arg-1, FIZZ1, Ym1/2	IL-10, TGF-β, CCL17, CCL22	Tissue remodelling	Human, mouse
M2b	IL-1β, LPS	IL-10^high^, IL-12^low^	IL-6, IL-10^high^, IL-12^low^, TNF-α	Immunoregulation	Human, mouse
M2c	IL-10, TGF-β, glucocorticoids	CD206 (human); Arg-1 (mouse)	IL-10, TGF-β, PTX3	Phagocytosis of apoptotic cells	Human, mouse
M2d	TLR + A_2_R ligands	IL-12^low^, TNFα^low^	IL-10, VEGF, iNOS	Angiogenesis, tumour progression	Mouse
M4	CXCL4	CD206, MMP7, S100A8	IL-6, TNF-α, MMP12	Weak phagocytosis, minimal foam cell formation	Human
Mox	Oxidized phospholipids	HMOX-1, Nrf2, Srxn1, Txnrd1	IL-1β, IL-10	Weak phagocytosis, proatherogenic	Mouse
HA-mac	Haemoglobin/Haptoglobin complex	CD163^high^, HLA-DR^low^	HMOX-1	Haemoglobin clearance, atheroprotective	Human
M(Hb)	Haemoglobin/Haptoglobin complex	CD206, CD163	ABCA1, ABCG1, LXRα	Haemoglobin clearance, high cholesterol efflux, atheroprotective	Human
Mhem	Haeme	ATF1, CD163	LXRβ	Erytrophagocytosis, atheroprotective	Human, mouse

* Table1 summarizes the main genes involved in macrophage differentiation/polarization.

ABCA1: ATP-binding cassette transporter A1; Arg-1: arginase-1; A_2_R: adenosine receptor A2; ATF1: cyclic AMP-dependent transcription factor 1; CD163: scavenger receptor for the haemoglobin–haptoglobin complex; CD206: mannose receptor; CCL17: chemokine (C-C motif) ligand 17; CXCL4: chemokine (C-X-C motif) ligand 4; FIZZ1: transcription factor found in inflammatory zone 1; HLA-DR: human leucocyte antigen DR; HMOX-1: haeme oxigenase 1; IFN-γ: interferon-γ; IL-1β: interleukin-1β; IL1RN: IL-1 receptor antagonist; iNOS: inducible nitric oxide synthase; LPS: lipopolysaccharide; LXRα: liver X receptor α; MMP7: matrix metalloproteinase 7; Nrf2: nuclear factor (erythroid-derived 2)-like; PTX3: pentraxin 3; ROS: reactive oxygen radical; S100A8: S100 calcium binding protein A8; Srxn1: sulfiredoxin 1; TGF-β: transforming growth factor β; TNF-α: tumour necrosis factor α; TLR: Toll-like receptor; Txnrd1: thioredoxin reductase 1; Ym1: T lymphocyte-derived eosinophil chemotactic factor.

Each M2 subset is characterized by a unique function. M2a macrophages express anti-inflammatory cytokines (IL-10 and TGF-β) and C-C motif chemokines CCL17 and CCL22 and contribute to tissue remodelling 20. Compared to human M2 macrophages, murine M2a macrophages express several signature markers such as chitinase 3-like 3 lectin (CHI3L1, also known as Ym1 or YKL-40), transcription factor found in inflammatory zone 1, arginase-1 and have developed arginine metabolism 21. M2b macrophages produce high amounts of IL-10 and low amounts of proinflammatory cytokines IL-1β, IL-6, IL-12 and TNF-α. This macrophage population possesses immunoregulatory properties 22. The M2c subset expresses IL-10, TGF-β, pattern-recognition receptor pentraxin-3 and high levels of Mer receptor kinase (MerTK) essential for efferocytosis 23. M2a, M2b and M2c macrophages were found in both humans and mice, whereas M2d subset was identified only in the mouse 24. This subset could be induced by treatment of M0 macrophages with Toll-like receptor agonists that activate the adenosine A2 receptor 25. Activation of the A2 receptor leads to the down-regulation of secretion of inflammatory cytokines TNF-α, IL-1β and IFN-γ and induces a unique proangiogenic activity in M2d macrophages associated with production of VEGF, nitric oxide and IL-10 26,27.

The treatment of M0 macrophages with haeme products could result in polarization to three phenotypically distinct types of macrophages such as HA-mac, M(Hb) and Mhem (Table1). HA-mac and M(Hb) populations that could be induced with the haemoglobin–haptoglobin complex were detected in humans 28,29. HA-mac macrophages express high levels of CD163, a scavenger receptor of the haemoglobin–haptoglobin complex and haeme oxygenase 1 (HMOX1) that is involved in haeme catabolism 30. M(Hb) subset highly expresses of scavenger receptors CD163 and CD206, liver X receptor α (LXRα) and ATP-binding cassette transporters ABCA1 and ABCG1. Therefore, M(Hb) macrophages have strongly regulated intracellular lipid balance and are stable to cholesterol uptake because of increased ABCA1- and ABCG1-mediated cholesterol efflux 31. In mice, haeme induces Mhem macrophages characterized with increased production of CD163, LXRβ, haeme-induced cyclic AMP-dependent transcription factor 1 (ATF1), HMOX1 and ABCA1 32. These macrophage subsets are busy with haemoglobin clearance in haemorrhagic sites. Furthermore, Mhem macrophages are able to engulf extravasated erythrocytes (eryhrophagocytosis) 33.

## Macrophage phenotypes in atherosclerosis

In the context of atherosclerosis, all subsets of macrophages mentioned above could be found in either human or murine plaques 34. Some of those play a proaherogenic role, whereas others contribute to atheroprotection. There are two macrophage populations (M1 and M2) that are commonly distributed in lesions. In plaques, proinflammatory M1 macrophages were found several decades ago, whereas M2 macrophages with anti-inflammatory properties were detected more recently 35. In humans, M2 macrophages positive for CD68 and CD206 were detected in the peripheral plaque regions with increased production of IL-4. These macrophages had reduced resistance against lipid accumulation because of low expression of LXRα, ABCA1 and ApoE, but showed high phagocytic activity 36. CD68^+^ CD206^+^ M2 macrophages are located in plaque regions enriched with iron suggesting about their role in iron recycling. Iron export by macrophages is controlled by LXRα that induces expression of ferroportin 37.

HA-mac, M(Hb) and Mhem macrophages could be found in haemorrhagic zones of unstable lesions where they phagocytize and utilize erythrocyte remnants and haemoglobin deposits. These macrophages are atheroprotective and resistant to transformation to foam cells because of high expression of nuclear receptors LXRα and LXRβ and transporters ABCA1 and ABCG1 responsible for cholesterol efflux 32.

In plaques, macrophage phenotype is subjected to the modulation by various factors including cytokines, chemokines, immune complexes and lipids (oxidized lipoproteins, cholesterol crystals and fatty acids) 38. In murine plaques, Kadl *et al*. 39 described phenotypically distinct macrophage subset called Mox. This proatherogenic subset is induced by oxidized phospholipids and protects from oxidative stress through nuclear factor (erythroid-derived 2)-like 2 (Nrf2)-mediated expression of antioxidant enzymes such as HMOX1, thioredoxin reductase 1 (Txnrd1) and sulfiredoxin-1 (Sxrn-1; Table1). In atherosclerotic lesions of low-density lipoprotein receptor (Ldlr)-deficient mice, Mox macrophages were shown to be widely presented accounting for 30% of all plaque macrophages, whereas M1 and M2 subsets accounted for 40% and 20% respectively 39.

Finally, CXCL4-induced M4 macrophages were observed in human lesions 40. Erbel *et al*. 41 reported identification of a small population of macrophages positive for matrix metalloprotease (MMP)-7 and Ca^2+^-binding protein S100A8, *e.g*. markers whose expression is induced by CXCL4 (Table1). This chemokine is released by activated platelets 42 and have effects on different classes of immune cells such as T cells, monocytes, macrophages, neutrophils and dendritic cells 43. In apoE-deficient mice, deletion of CXCL4 results in the decrease in plaque size indicating the proatherogenic effects of this chemokine 44. Probably, M4 macrophages may play a proatherogenic role as they express some proinflammatory cytokines such as IL-6 and TNF-α and do not express a scavenger receptor CD163 required for the induction of atheroprotective HMOX1 16,45. During polarization to M4 macrophages, CD163 expression is down-regulated by CXCL4 16. In M4 macrophages, phagocytic activity is almost completely suppressed 40.

## Changes in macrophage transcriptome during differentiation and polarization

### Monocyte–macrophage differentiation

Differentiation of monocytes to M0 macrophages driven by M-CSF is accompanied by significant changes in gene expression profiles. As mentioned above, most changes in cultured monocytes happen during the first 3 days even expression of some genes could return to the initial level 11. Using a microarray analysis, Martinez *et al*. 11 showed significant (>3 times) changes in expression levels of 868 transcripts (2.2% of a total transcriptome) during monocyte–macrophage differentiation. Of them, expression levels of 390 (1%) genes were changed only during the differentiation process, but returned to basal levels in completely differentiated macrophages. Those include activation of expression of cell cycle regulators such as cyclins and cell division-associated proteins 1, 2, 5, 6, 7 and 20 11, with three main nodes of activated genes clustered around cyclin-dependent kinase 1, chemokine (C-C motif) ligand 2 (CCL2) and Bcl-2-like protein 11 (BCL2L11) and one cluster associated with the down-regulation of human leucocyte antigen (HLA) genes. Notably, these activated genes were enriched with those encoding nuclear proteins, but contained the low number of soluble factors. This is a characteristic of the differentiation process and reflects activation of genes essential only for differentiation.

Martinez *et al*. 11 found the second cluster of 478 genes (1.2%) with significant changes in the expression during differentiation that maintained in mature macrophages and were resistant to polarizing signals. In this cluster, the number of nuclear factors was substantially decreased, whereas a marked increase in the number of genes encoding soluble factors and membrane receptors. These genes are stably regulated and are likely to be involved in the induction and maintenance of macrophage-specific features. Interactome analysis revealed three main nodes clustered around the IL-1β, IL-8 and ApoE genes, *e.g*. genes that play a crucial role in macrophage maturation and function.

Analysis of functional categories that significantly change during monocyte–macrophage differentiation showed the most marked alterations in expression of genes involved in lipid metabolism 11. Lipid metabolism plays a critical role in macrophage function and life as macrophages respond to and secrete a wide range of bioactive lipid products 38. In particular, the differentiation affects expression profiles of enzymes involved to the biosynthesis of eicosanoids and associated with gradual decrease of prostaglandin-endoperoxide synthases 1 and 2, leucotriene A4 hydrolase and arachidonate 5-lipoxygenase. Probably, down-regulation of enzymes involved in eicosanoid production during macrophage maturation reflects inactive status of M0 macrophage. However, synthesis of eicosanoids could be restored upon macrophage activation 46,47.

After treatment of a human monocyte cell line U937 with phorbol 12-myristate 13-acetate (PMA) followed activation with LPS, Baek *et al*. 48 observed differentiation of U397 cells to macrophages characterized by proinflammatory phenotype. At 24 hrs of PMA-directed differentiation, significant changes in expression of a total of 595 genes (up-regulation) and 324 genes (down-regulation) were detected. After 2 hrs of subsequent LPS activation, 278 additional showed increased expression, whereas the expression of 107 additional genes was significantly reduced 48. Among top 50 up-regulated genes during monocyte–macrophage differentiation, there were many transcription factors that control the differentiation process including early growth response protein 1 (EGR1), EGR2, V-maf musculoaponeurotic fibrosarcoma oncogene homolog B, H2.0 homeobox protein (HLX), transcription factor 7-like 2 and myocyte enhancer factor-2 (MEF2) family of transcription factors, Krüppel-like Factor 2 (KLF2) and KLF4. KLF2 and KLF4 were described to be involved in both macrophage differentiation and proinflammatory activation 49,50. Baek *et al*. 48 also observed significant up-regulation of several negative transcriptional regulators such as inhibitor of DNA binding 3 (ID3), B-cell lymphoma 6 protein (BCL6), nuclear factor IL-3 regulated and histone deacetylases (HDAC) 3 and 7/9 that control terminal differentiation of myeloid cells and prevent excessive proinflammatory response 51,52. HDAC7 and HDAC9 were shown to form repression complexes with the MEF2 family of transcription factors thereby preventing further differentiation 53.

Interestingly, Das *et al*. 49 showed that expression of KLF2 is down-regulated during differentiation of THP-1 monocytes to macrophages and subsequent proinflammatory activation of macrophage. In contrast, KLF2 was up-regulated during differentiation of U937 cells 48. The discrepancy of these results could be probably explained by a less mature stage of THP-1 cells compared to U937 cells 49. U937 cells express CD14, a marker of both monocytes and macrophages and CD11b, a macrophage marker 48,54. THP-1 cells highly express CD14, but almost luck expression of CD11b, which indeed suggest about a less maturity of these cells towards macrophages 55,56.

Eijgelaar *et al*. 57 showed high similarities in the expression pattern of human resident alveolar, splenic and plaque macrophages and Kupffer cells by identifying a subset of 500 genes with equivalent expression levels. Those include CD68 (a surface receptor for LDL), IL-1 receptor antagonist, CD1c (a major histocompatibility complex (MHC) class I-like molecule expressed on the surface of antigen-presenting cells) and other genes. However, most of those 500 genes were involved in standard cellular processes such as protein synthesis, transcription and phosphorylation. These data suggest for high conservation of expression profiles of genes in macrophages of different tissue location involved in maintaining of key intracellular mechanisms.

### ‘Classical’ polarization of M0 macrophages to M1

Martinez *et al*. 11 reported significant changes in expression of 2053 transcripts (5.2% of a total transcriptome) during proinflammatory activation of murine M0 macrophages to M1 macrophages with LPS + IFN-γ. Among those, 1108 (2.8%) genes were specifically associated with classical macrophage activation. Those include up-regulation of several transcription factors such as homeobox expressed in ES cells 1, IFN regulatory factors 1 and 7, ATF3, nuclear receptor 4A1 (NR4A1), signal transducer and activator of transcription STAT3 and nuclear factor NF-κB. In macrophages, NR4A1 could be specifically induced by LPS through NF-κB-mediated mechanism as the promoter of the NR4A1 gene was shown to contain two highly conserved NF-κB responsive elements 58. NF-κB is essential for the activation of expression of genes whose products are involved in the induction of the proinflammatory response. NR4A1 acts synergistically with NF-κB potentiating the induction of inflammatory gene expression in response to LPS 59. Those include a broad spectrum of chemokines (CXCL9, CXCL10, CXCL11, CCL5, CCL15 and CCL19), proinflammatory cytokines (IL-6, IL-12B, IL-15 and TNF-α) and immune receptors [TNF ligand superfamily 10 (TNFSF10 or TRAIL), C-C chemokine receptor 7 (CCR7), IL-2 receptor α chain, IL-15 receptor α chain and IL-7 receptor] that characterize the proinflammatory phenotype of macrophages 22. Furthermore, the expression of the NR4A subfamily of orphan nuclear receptors was found in human plaque macrophages suggesting for their involvement in atherosclerosis-associated inflammation 60.

Proinflammatory polarization of murine macrophages towards the M1 type is accompanied with the induction of arginase-2 11, a mitochondrial enzyme that is involved in metabolism of L-arginine. L-arginine is necessary for the expression of the activated macrophage cytotoxic effector mechanism 61. In human macrophages, M1 polarization is also characterized by the activation of eicosanoid production and signalling that is reflected by marked up-regulation of cyclooxygenase-2 62.

### ‘Alternative’ polarization of M0 macrophages to M2

As shown by Martinez *et al*. 11, IL-4-dependent polarization of murine macrophages to the M2 type significantly affects expression of the less number of genes (104 or 0.3%) compared to M1 polarization. The range of genes whose expression changes in M2 macrophages is especially enriched with those involved in the immune function including chemokines CCL13, CCL17, CCL18 and CCL23 and indoleamine-pyrrole 2,3-dioxygenase (IDO), an immunomodulatory enzyme. Ectopic expression of IDO in THP-1 cells results in expression of M2 markers such as IL-10 and CXCR4 and decreases the M1 markers such as CCR7 and IL-12 α-chain 63. In addition, the expression of IDO was shown to be associated with the induction of anti-inflammatory and immunoregulatory properties in macrophages, a characteristic of the M2 type 63.

Compared to M1 macrophages, murine M2 cells have strongly up-regulated the expression of a range of surface markers such as CD36 (a multiligand-binding scavenger receptor), CD209/DC-SIGN (a C-type lectin receptor that binds mannose type carbohydrates), CD163, CD206, a lectin C-like receptor CLEC7A/Dectin-1 (a pattern-recognition receptor (PPR) that senses a variety of fungal and plant glucans 64, and membrane-spanning 4-domains (MS4A) A4 and MS4A6A, two CD20-like receptors probably involved in the control of cell cycle of haematopoietic cells 65,66. Compared to M1, murine M2 macrophages showed substantially increased the expression of several transcription factors such as c-MAF, EGR2 and growth arrest-specific 7 (GAS7) 11. c-MAF is a the basic leucine zipper family of transcription factors, which is expressed in monocytes and macrophages and required for the anti-inflammatory polarization of macrophages as it inhibits expression of proinflammatory IL-12 and stimulates production of anti-inflammatory IL-10 67–69. GAS7 was shown to play a role in the control of macrophage metabolic network and is associated with metabolic traits such as obesity in humans and mice 70. However, up-regulation of EGR2 is dispensable for M2 polarization because this factor is involved in the regulation of expression of genes involved in the growth and differentiation of many cell types. Furthermore, knockout of EGR2 in mice does not impair function and differentiation of macrophages 71.

### CXCL4-induced M4 macrophages

Compared to M-CSF, which primes monocyte differentiation to M0 macrophages, CXCL4 induces a unique transcriptome in M4 macrophages. Compared to M0 macrophages, Gleissner *et al*. 40 found 375 genes differentially expressed in M4 macrophages, with 206 up-regulated and 169 down-regulated genes. Among immune-related genes related, M4 macrophages have increased expression of CCL18 and TRAIL. In addition, M4 cells had elevated the expression of genes such as HLA class II involved in antigen presentation. In M0 macrophages, the expression of genes involved in chemotaxis (CCL3, CCL7 and CCR1) and cell adhesion (integrins ITGAV and ITGA6) was significantly up-regulated.

In overall, the transcriptome of M4 macrophages is not clearly pro- or anti-atherogenic but is distinct from that of M1 and M2 subsets 40. Between M0 and M4 macrophages, there were no differences in expression levels of proinflammatory cytokines IL-1β, IL-8 and IL-12. However, the expression of M1-specific (IL-6 and TNF-α) and M2-specific (CCL18 and CCL22) markers was higher in M4 cells then in M0 macrophages. The expression of MMP7 and MMP12 was higher in M4 cells, whereas MMP8 expression was increased in M0 macrophages.

M4 macrophages express low levels of scavenger receptors essential for uptake of modified lipids, but surprisingly high levels of the cholesterol efflux transporter ABCG1. Therefore, compared to M0, M4 cells are relatively resistant to transformation to foam cells. However, the expression levels of atheroprotective apolipoproteins such as ApoE and ApoC1 are lower in M4 cells compared with M0 40. These data suggest that CXCL4 alone is unlikely to be sufficient to promote atherosclerosis through the induction of M4 macrophages because some atherosclerosis-related genes are up-regulated in M4 macrophages, whereas other genes are down-regulated. Proatherogenic activity of CXCL4 is likely to be multifactorial, including its effects on endothelial cells and different types of leucocytes. Although M4 macrophages could be found in atherosclerotic plaques, their role in atherosclerosis remains unclear and hence should be evaluated.

### Cholesterol load

In atherosclerosis, macrophages take up modified low-density lipoproteins (LDL) with help of scavenger receptors. While modified LDL degrade in lysosomes, free cholesterol is transferred from lysosomes to the endoplasmic reticulum (ER). In ER, cholesterol is esterified and then store in lipid droplets 72. Lipid droplets could be delivered back to lysosomes *via* the mechanism of autophagy where cholesterol esters transform to free cholesterol by lysosomal acid lipase 73. Free cholesterol could leave cytoplasm through passive diffusion or through efflux by membrane transporters ABCA1, ABCG1 or scavenger receptor B1 (SCARB1) 74,75. However, as expression of SCAR is not regulated by intracellular cholesterol content, macrophages cannot limit cholesterol accumulation and transform to foam cells 76.

Cholesterol load is accompanied by significant changes in the macrophage transcriptome. Berisha *et al*. 77 studied expression of genes in cholesterol-loaded macrophages derived from atherosclerosis-prone DBA/2 ApoE-deficient mice and from atherosclerosis-resistant AKR ApoE-deficient mice. DBA/2 ApoE-deficient animals were shown to develop lesions whose average size is 10 times larger than that in AKR ApoE-deficient mice 78. In response to cholesterol loading, Berisha *et al*. 77 showed significant changes in the expression levels of 567 transcripts between DBA/2 and AKR macrophages, with 236 up-regulated and 331 down-regulated transcripts. The most significant changes were observed in genes whose products were involved in the lysosomal function and ER stress suggesting that these pathways are primarily affected with cholesterol load 77. Among lysosomal genes, there were various enzymes including proteases cathepsins CtsC, CtsE and CtsZ, and those involved in lipid metabolism such as palmitoyl-protein thioesterase Ppt1 and ceramidase Asah1. Mutations in the human Asah1 genes were shown to cause Farber disease, a lipid storage disorder 79. Among cholesterol-regulated lysosomal genes, there were adapter-related protein complexes Ap3d1, Ap3m2 and Ap1s1, which are involved in the transport between lysosomes and ER 80, sortilin 1 and mucolipin 1. Sortilin 1 was shown to contribute to hepatic apoB lipoprotein secretion and LDL intake 81. Mucolipin 1 is involved in lipid transport from lysosome 82.

Among top 10 genes whose expression was significantly changed in response to cholesterol loading, there were three genes (CHOP, Trib3 and Arf4) that contribute to the ER stress. The CHOP gene encoding C/EBP homologous protein was up-regulated in AKR macrophages, but down-regulated in DBA/2 macrophages 77. CHOP is a transcription factor that is activated in ER stress and unfolded protein response (UPR) 83. Tabas 84 showed that free cholesterol could cause ER stress in macrophages associated with CHOP induction and increased apoptosis. The Trib3 gene was up-regulated in APK macrophages 77. This gene encodes tribbles homolog 3, a pseudokinase that is implicated in ER stress-induced apoptosis 85. Inactivation of Trib3 in mice deficient for Ldlr and ApoE was shown to lead to smaller aortic plaques suggesting for the proatherogenic role of this prot-ein 86. Finally, the ADP-ribosylation factor 4 (Arf4) gene whose expression was up-regulated in AKP macrophages 77 encodes small guanine nucleotide-binding protein. Recently, Reiling *et al*. 87 reported that ARF4 activation is related to ER stress and apoptosis as Arf4 depletion is associated with increased resistance to ER stress, cell viability and Golgi integrity.

Indeed, accumulation of cholesterol in macrophages leads to the up-regulation of ER stress-related genes and is likely to increase macrophage apoptosis through the induction of chronic ER stress and acute UPR. However, increased apoptosis of macrophages caused by cholesterol-induced ER stress could be atheroprotective at early atherosclerosis stages as it activates phagocytosis of apoptotic cells and attenuated plaque growth 84. In addition, ER stress could stimulate the protective mechanism of autophagy associated with elevated lysosomal hydrolysis of cholesterol esters to free cholesterol that is subjected to further efflux 73. However, in advanced atherosclerosis, increased macrophage death contributes to atherogenesis because of decreased clearance of dead cells in the necrotic plaque core.

### Mox macrophages

As mentioned above, proatherogenic oxidized phospholipids induce murine macrophage polarization to a novel phenotypically distinct subset termed Mox macrophages 39. Mox macrophages are markedly different from M1 and M2 macrophages. Furthermore, the treatment of either M1 or M2 subtype with oxidized phospholipids causes phenotypic switch towards Mox. Compared to M1 and M2, phagocytic activity of Mox macrophages is impaired 39.

The treatment of M0 macrophages with oxidized phospholipids results in the induction of a unique transcriptome. In Mox macrophages, Kadl *et al*. 39 showed significant changes in expression levels of 119 genes after treatment with oxidized phospholipids. Of those, only 38 genes were up-regulated in both Mox and M1 macrophages after incubation with oxidized phospholipids. Only 13 genes were overlapping between M2 and Mox subsets. Genes that were exclusively activated in Mox macrophages include a set of redox-regulated genes such as HMOX-1, Txnrd1, Sxrn-1, regulatory (Gclm) and catalytic (Gclm) subunits of glutamate-cysteine ligase, a rate-limiting enzyme of glutathione biosynthesis 88 and other genes such as nuclear receptor 4A2 (Nr4A2 or Nurr1), Vegfa and Trib3. Transcription factor Nurr1 could be induced in macrophages in response to inflammatory signals and leads to the inhibition of expression of proinflammatory genes through suppressing the activity of NF-κB 58. In addition, this factor plays the atheroprotective role by decreasing lipid loading in plaque macrophages 89. On the other hand, expression of Vegfa could suggest for a putative involvement of Mox macrophages to intraplaque neovascularization, whereas up-regulation of Trib3 could indicate increased vulnerability to ER stress induced by oxidized phospholipids.

Oxidized phospholipids could recruit monocytes/macrophages by binding to the chemokine receptor Ccr2 90 and subsequent activation of redox-regulated transcription factor Klf2 38. In pro-oxidant conditions, Klf2 is activated by dissociation from its negative regulator Kelch-like ECH-associated protein 1 (Keap1) and moves to the nucleus where it primes expression of antioxidant and detoxifying genes such as HMOX-1, Txnrd1, glutathione S-transferase, Gclm and Gclm, all of which are Mox-specific markers 91. Expression of Klf2 is stimulated by oxidative stress as the Klf2 promoter contains so called the antioxidant response element 92. Indeed, this factor is a key master regulator of macrophage polarization to Mox phenotype. These data suggest for atheroprotective properties of Mox macrophages which contribute to the regulation of intraplaque redox status. However, the suppressed phagocytic activity of Mox macrophages could potentially enhance plaque progression and destabilization 39. Therefore, Mox macrophages represent a distinct cell subset that exhibits no clear pro- or anti-inflammatory activity compared to M1 and M2. A precise role of this macrophage subpopulation in atherosclerosis is still unknown and should be further investigated.

## Conclusion

In conclusion, we see that macrophages have a plasticity to change the phenotype in response to various stimuli. The polarization is accompanied with significant changes in the macrophage transcriptome. The polarization of M0 macrophages to the ‘classical’ proinflammatory M1 subset looks as the most profound, with changes in the expression of more than 1500 transcripts 11,39. In contrast, the anti-inflammatory M2 macrophages such as the alternative M4 and Mox subsets show significant changes in the expression of the less number of genes compared to M1. That could suggest that polarization to the M1 subtype could represent the commonest proinflammatory mechanism of activation of macrophages. Other subsets such as M2, M4 and Mox are less frequent and therefore are the uncommon pathway of the macrophage differentiation/polarization.

However, there are no whole-genome transcriptome analysis of haeme-induced macrophages subsets such as HA-mac, M(Hb) and Mhem have been performed. In atherosclerosis, these macrophages play the atheroprotective role, especially in vulnerable plaques that have recurrent haemorrhage events. Therefore, the expression signatures of these macrophage subpopulations should be studied in atherosclerotic lesions. It is also necessary to evaluate the role of M4 and Mox subsets because their concrete involvement in atherogenesis remains unclear. It is likely that these two macrophage phenotypes may be either atheroprotective or proatherogenic depending on the stimuli.

Significant heterogeneity observed in macrophage populations after polarization and absence of consensus among immunologists on how to define macrophage activation seriously hampers macrophage classification and nomenclature. However, recently, researchers attempted to define standards essential for description of macrophage activation on the basis of three principles such as source of macrophages, designation of stimulators and collection of markers specific for each type of activation 93. To date, it remains unknown how macrophages are heterogeneous. New phenotypically distinct subsets are likely to be found in the future. Indeed, defining standards for already existing macrophage subsets should help in identification of novel macrophage populations.
